# In Vitro Evaluation of Electroactive GelMA/PCL/Graphene Oxide Scaffolds for Peripheral Nerve Repair

**DOI:** 10.3390/jfb17080376

**Published:** 2026-08-03

**Authors:** Yingcong He, Ruiqiang Chen, Shuangqi Gao, Jie Pan, Wei Zhang, Shulin Bai, Peixun Zhang, Xingxing Fang

**Affiliations:** 1 Department of Spine Surgery, The Third Affiliated Hospital of Sun Yat-sen University, Guangzhou 510630, China; 2 Guangdong Provincial Key Laboratory of Stomatology, Hospital of Stomatology, Guanghua School of Stomatology, Sun Yat-sen University, Guangzhou 510055, China; 3 Departments of Neurosurgery, The Third Affiliated Hospital of Sun Yat-sen University, Guangzhou 510630, China; 4 Department of Pathology, Stanford University School of Medicine, Stanford, CA 94305, USA; 5 School of Materials Science and Engineering, Peking University, Beijing 100871, China; 6 Department of Orthopedics and Trauma, Peking University People’s Hospital, Beijing 100044, China; 7 Department of Orthopedics and Trauma, Peking University People’s Hospital Qingdao Hospital, Qingdao 266111, China

**Keywords:** GelMA, PCL, graphene oxide, electrospinning, peripheral nerve repair, conductive scaffold, electrical stimulation

## Abstract

This study developed electroconductive GelMA/PCL/graphene oxide (GO) nanofibrous scaffolds and performed a preliminary in vitro evaluation for peripheral nerve repair, focusing on optimizing the GO concentration. GO (0.1–1.0 wt%) was incorporated into GelMA/PCL via electrospinning. The 1.0 wt% GO scaffold exhibited reduced fiber diameters (200–300 nm) and enhanced electrical conductivity (8.4 × 10^−4^ mS/cm). All scaffolds were superhydrophilic (contact angle ≈ 0°). Tensile strength and fracture strain increased up to 0.5 wt% GO but decreased at 1.0 wt% GO to levels not significantly different from those of the control. PC12 cells demonstrated robust adhesion, proliferation, and viability on all scaffolds. Under electrical stimulation (100 mV/cm), the 1.0 wt% GO scaffold promoted qualitative axonal elongation and directional alignment compared with scaffolds lacking GO or subjected to no electrical stimulation. Scaffold-immobilized GO showed no cytotoxicity in CCK-8 assays, indicating a favorable preliminary safety profile. These results highlight the potential of GelMA/PCL/1.0 wt% GO scaffolds as a promising platform for advanced nerve guidance conduits; however, further validation using primary neurons and in vivo models is required.

## 1. Introduction

Peripheral nerve injury (PNI) is a major global health challenge that frequently results in deficits in sensory and motor functions, the onset of persistent neuropathic pain, and associated psychological disorders, significantly diminishing patients’ quality of life [1,2]. Currently, clinical management of traumatic PNI includes surgical intervention, nerve autograft transplantation, and physical rehabilitation protocols intended to promote functional recovery [2,3]. However, these therapeutic interventions are limited by several drawbacks, such as donor site complications and mismatches in nerve dimensions [4,5]. Consequently, there is an urgent need for innovative therapeutic strategies that can overcome these obstacles and improve patient outcomes.

With the advancement of tissue engineering, various biocompatible materials have been employed to develop nerve guidance scaffolds (NGSs) as a promising alternative to autografts [6,7,8]. A wide range of synthetic and natural polymers have been explored, including polycaprolactone (PCL), poly (lactic-co-glycolic acid) (PLGA), gelatin, chitosan, and silk fibroin, each offering distinct advantages in terms of biodegradability, mechanical properties, and cellular compatibility. Many researchers are currently focusing on designing nerve guidance conduits (NGCs) with improved physiological functionality through the strategic selection and modification of biomaterials, as well as the integration of emerging technologies [6,8,9,10].

A critical advancement in NGC design has been the incorporation of conductive materials to recapitulate the electrophysiological microenvironment of native neural tissue. Carbon-based nanomaterials, including carbon nanotubes, graphene, graphene oxide (GO), and reduced graphene oxide (rGO), have been extensively investigated for neural tissue engineering applications [11,12,13]. Among these, rGO has been widely favored for its high electrical conductivity; however, its hydrophobic nature and the harsh chemical reduction processes required for its production may compromise bioactivity and introduce cytotoxic residues. In contrast, GO maintains oxygen-rich functional groups (epoxy, hydroxyl, and carboxyl) that confer superior aqueous dispersibility, enhanced hydrophilicity, and the capacity for non-covalent biomolecular interactions—properties that are particularly advantageous for creating a cell-supportive microenvironment [14,15]. Furthermore, the moderate conductivity of GO, while lower than that of rGO, may be physiologically appropriate, as excessive electrical conductivity in tissue scaffolds could potentially lead to undesirable current leakage or electrochemical byproduct formation [16].

Although both studies utilize GelMA/PCL as the base biomaterial matrix, their underlying scientific objectives diverge significantly: the prior study prioritized optimization of electrical conductivity, whereas the present work investigates graphene oxide’s (GO) oxygen-rich surface chemistry as a bioactive signaling cue—wherein moderate conductivity is deliberately retained to establish a safer and more physiologically representative electroactive microenvironment. A direct experimental comparison between GO and reduced graphene oxide (rGO) was not conducted; this constitutes a key avenue for future investigation.

Our group previously reported rGO-GelMA-PCL hybrid nanofibers for peripheral nerve regeneration, demonstrating the feasibility of this composite system [10]. The present study builds upon this foundation. We hypothesize that the oxygen-containing groups on GO can participate in protein adsorption and cell–matrix interactions, providing a biochemically instructive substrate that complements electrical stimulation.

To test this, we fabricated a series of GO-reinforced GelMA/PCL scaffolds with precisely controlled GO concentrations (0–1.0 wt%) and conducted comprehensive physicochemical and biological evaluations. Direct experimental comparison of GO vs. rGO in terms of protein adsorption or biochemical signaling is beyond the scope of this work and will be pursued in future studies.

Electrospinning technology serves as a crucial and highly promising approach in the field of tissue engineering. When combined with other nontoxic materials designed to mimic natural tissues, electrospun composites can generate fibrous architectures spanning from the nanoscale to the microscale, featuring interconnected porous structures that closely resemble the extracellular matrix (ECM) found in natural biological tissues [17]. These electrospun scaffolds not only offer structural support but also establish a microenvironment that promotes cell adhesion, proliferation, and differentiation. To date, electrospun constructs have been extensively investigated for their applications in tissue engineering, particularly in the regeneration of bone, muscle, cartilage, skin, nerve, and vascular tissues [18,19,20,21].

The incorporation of graphene oxide (GO) into the polymer matrix can modulate the physicochemical properties of electrospun nanofibrous scaffolds used in tissue engineering, owing to its capacity to mimic the composition of the extracellular matrix [16,17]. Being oxygen-rich, GO is inherently hydrophilic and readily disperses in water, thereby promoting cell growth, enhancing drug delivery, and imparting antimicrobial properties to electrospun nanofiber matrices [14]. Moreover, graphene oxide-based scaffolds can enhance the recovery of electrical nerve signals due to their excellent conductivity and electrical stability [16,21,22]. Although the restoration of bioelectric signaling is essential for peripheral nerve regeneration [23,24], the optimization of electrical stimulation protocols for tissue engineering scaffolds remains an active area of research.

Furthermore, it is critical to distinguish between the cytotoxicity profiles of dispersed GO nanoparticles and scaffold-immobilized GO. Numerous toxicological studies have demonstrated that GO nanoparticles can induce dose-dependent cytotoxicity when directly exposed to cells in suspension, primarily through membrane disruption, oxidative stress, and inflammatory responses [25,26]. However, when GO is firmly embedded within a crosslinked polymer matrix, as in the electrospun scaffolds described herein, the exposure of cells to free GO nanosheets is substantially minimized. The polymer encapsulation restricts direct physical contact between GO and cellular membranes while preserving the bulk conductivity and surface functionality benefits of the incorporated GO. This distinction is essential for understanding the favorable biocompatibility observed in GO-based composite scaffolds despite reports of GO nanoparticle toxicity [27,28].

This study aimed to fabricate a series of graphene oxide (GO)-reinforced GelMA/PCL composite scaffolds via electrospinning, followed by preliminary evaluation of their physicochemical properties and in vitro biocompatibility. The results indicate that the GelMA/PCL scaffold incorporating 1.0 wt% GO exhibits superior conductivity and supports neurite outgrowth under electrical stimulation, demonstrating significant potential as a candidate for neural tissue engineering.

## 2. Materials and Methods

### 2.1. Materials

Graphene oxide (GO) was purchased from Tanfeng Tech. Inc. (Suzhou, China). According to the manufacturer’s specifications, the GO had a lateral size distribution of 0.5–5 μm, a thickness of 0.5–3.0 nm (1–2 atomic layers). The GO contained abundant epoxy, hydroxyl, and carboxyl functional groups. Polycaprolactone (PCL, Mn = 80,000 g/mol) and type A gelatin (bloom strength ~300 g) were purchased from Sigma-Aldrich (St. Louis, MO, USA). Irgacure 2959 (2-hydroxy-4′-(2-hydroxyethoxy)-2-methylpropiophenone) was obtained from BASF (Ludwigshafen, Germany). All other reagents were of analytical grade and used as received.

### 2.2. Synthesis of Gelatin Methacryloyl (GelMA)

GelMA was synthesized by reacting type A gelatin with methacrylic anhydride (MA) following a previously established protocol. Briefly, 10 g of gelatin was dissolved in 100 mL of phosphate-buffered saline (PBS, pH 7.4) at 50 °C under vigorous stirring. Methacrylic anhydride (8 mL) was added dropwise at a rate of 0.5 mL/min, and the reaction proceeded for 3 h at 50 °C. The mixture was then diluted with 400 mL of warm PBS (40 °C) and dialyzed against distilled water using 12–14 kDa cutoff dialysis tubing for 7 days at 40 °C, with water changed twice daily. The resulting GelMA solution was filtered, lyophilized, and stored at −20 °C.

### 2.3. Fabrication of GelMA/PCL/Graphene Oxide Scaffolds

GO powder was first dispersed in 2,2,2-trifluoroethanol (TFE) at predetermined concentrations (0.1, 0.3, 0.5, and 1.0 wt% relative to total polymer mass) and ultrasonicated (Sonics VCX 750, 40% amplitude; Sonics & Materials, Inc., Newtown, CT, USA) for 30 min in an ice bath to obtain homogeneous suspensions. Subsequently, 10 g PCL and 10 g GelMA were gradually added to the GO-TFE solution under continuous stirring at 40 °C for 2 h. The pH of the solution was adjusted to 4.5–5.0 using 0.1 M HCl, as alkaline conditions were found to induce GelMA aggregation and phase separation, resulting in an incompatible suspension unsuitable for electrospinning. The photoinitiator Irgacure 2959 was added at 0.5% (*w*/*v*) relative to the total solution volume. The final polymer concentration was maintained at 20% (*w*/*v*).

During the electrospinning process (equipment: IonBeam WL-2C, Beijing, China), the following parameters were kept constant: flow rate, 2 mL/h; distance between the needle tip and the collector, 20 cm; applied voltage, 15 kV; ambient temperature, 30–40 °C; relative humidity, 30–40%. The collector rod rotated at a speed of 1000 rpm. UV crosslinking was performed in situ during electrospinning using a 365 nm UV light source at an intensity of 9 W/cm^2^. The exposure time was approximately 2–4 s per fiber segment as it traveled from the needle tip to the collector. Complete crosslinking was verified by the formation of a water-insoluble fibrous mat after immersion in PBS at 37 °C for 24 h. The GelMA/PCL nanofiber scaffold (without GO) was fabricated using the same procedure as a control. After electrospinning, all scaffolds were dried under vacuum for a minimum of three days to remove residual solvent.

### 2.4. Characterization of Scaffolds

#### 2.4.1. Scanning Electron Microscopy (SEM)

Scanning electron microscopy (JEOL JSM-7900F, Tokyo, Japan) was used to examine the surface morphology of the electrospun nanofibers at an accelerating voltage of 10 kV. A thin layer of gold was sputter-coated onto the scaffolds prior to imaging. The average nanofiber diameter was calculated based on 48 random measurements performed using ImageJ v2.1.4.7 software on the obtained SEM images from six independent regions per sample.

#### 2.4.2. Fourier Transform Infrared Spectroscopy (FTIR)

FTIR spectroscopy (Bruker Tensor 27, Ettlingen, Germany) was employed to confirm the chemical composition of the GelMA/PCL/GO scaffolds and to verify successful incorporation of GO. Spectra were recorded in attenuated total reflectance (ATR) mode over the range of 4000–600 cm^−1^ with a resolution of 4 cm^−1^ and 64 scans per sample.

#### 2.4.3. Raman Spectroscopy

Raman spectroscopy (Horiba LabRAM HR Evolution, Villeneuve-d’Ascq, France) was used to confirm the presence of GO within the nanofibrous scaffolds. Spectra were acquired using a 532 nm laser excitation source with a 50× objective lens. The G-band (~1590 cm^−1^) indicates the structural features of GO within the polymer matrix.

#### 2.4.4. Thermogravimetric Analysis (TGA)

Thermogravimetric analysis (TGA-DSC-DTA, Q600 SDT, New Castle, DE, USA) was performed to quantify the actual GO content in the composite scaffolds and assess thermal stability. Samples (approximately 5 mg) were heated from 30 °C to 800 °C at a rate of 10 °C/min under a nitrogen atmosphere.

### 2.5. Electrical Conductivity Measurements

The electrical properties of the GelMA/PCL/GO scaffolds were characterized using both cyclic voltammetry (CV) and electrochemical impedance spectroscopy (EIS) on an electrochemical workstation (Bio-Logic, Seyssinet-Pariset, France). For CV measurements, scaffolds were cut into rectangular samples (30 mm × 10 mm) and mounted in a three-electrode configuration with the scaffold serving as the working electrode, a platinum wire as the counter electrode, and an Ag/AgCl electrode as the reference. CV curves were recorded at a scan rate of 50 mV/s over a potential window of −0.5 to +0.5 V. The electrical conductivity σ (S/cm) was calculated from the measured resistance using σ = L/(R × S), where L is the sample length (cm), R is the resistance (Ω), and S is the cross-sectional area (cm^2^). The impedance data were fitted to an equivalent circuit model to extract the bulk resistance. Six replicate samples were measured per group.

### 2.6. Water Contact Angle Measurements

Water contact angle (WCA) measurements were performed to evaluate the hydrophilicity of GelMA/PCL/GO scaffolds using a video contact angle measurement system (Dataphysics DCAT21, Filderstadt, Germany). A 5 μL water droplet was gently deposited onto the surface of the electrospun nanofibrous scaffolds. Measurements were recorded within 5 s of droplet deposition, and the contact angle was determined using the sessile drop method. Five measurements were performed per sample.

### 2.7. Degradation Test

The in vitro degradation behavior of the hybrid scaffolds was assessed under two conditions: (1) enzymatic degradation in phosphate-buffered saline (PBS, pH 7.4) containing 0.5 U/mL collagenase type II solution (Life Technologies, Catalog No. 17101-015, USA), and (2) hydrolytic degradation in PBS (pH 7.4) without enzyme. Pre-weighed scaffolds were immersed in the respective media and incubated in a temperature-controlled shaking water bath at 37 °C for a period of 4 weeks. The immersion medium was replaced every 3 days. At each designated time point (days 1, 3, 7, 14, 21, and 28), the samples were collected, rinsed three times with ultrapure water, lyophilized, and subsequently reweighed. The weight loss was calculated using the following formula:

Weight loss (%) = [(M_0_ − M_t_)/M_0_] × 100%

M_0_ represents the initial weight of the sample, while M_t_ denotes the residual weight of the sample collected at each time point. Data for each time point were obtained from six replicate samples per group.

Statistical analysis was performed using two-way repeated measures ANOVA with Tukey’s post hoc test (see Results).

### 2.8. Mechanical Characterization

The mechanical properties of the GelMA/PCL/GO scaffolds were evaluated using a biomaterials testing instrument (CARE, IBTC-5000, Tianjin, China) under a constant tensile strain rate of 0.1 mm/min in ambient conditions. All scaffold samples were fabricated in a dumbbell shape with rectangular dimensions of 13 × 5 mm^2^ (length × width) and an average thickness of approximately 0.07 mm, as measured by a digital micrometer. A minimum of six samples were tested per group. The mechanical parameters, including elastic modulus, ultimate tensile strength, and fracture strain, were calculated from the stress-strain curves.

### 2.9. Cell Culture and In Vitro Electrical Stimulation

Prior to cell seeding, all scaffolds were sterilized by immersion in 70% ethanol for 2 h, followed by three washes with sterile PBS and overnight incubation in complete culture medium. Rat pheochromocytoma PC12 cells were obtained from Biospecies (Guangzhou, China). Cells were maintained in RPMI-1640 medium supplemented with 10% horse serum, 5% fetal bovine serum, and 1% penicillin–streptomycin in a humidified incubator at 37 °C with 5% CO_2_. PC12 cells were seeded onto the scaffolds at a density of 3 × 10^4^ cells/cm^2^ in a 12-well plate and incubated for 24 h to allow cell attachment before the application of electrical stimulation (ES).

A direct current power supply was utilized to generate a stable electric field of 100 mV/cm, which was delivered through two parallel platinum electrodes inserted into the cell culture plate. The electric field strength of 100 mV/cm was selected based on prior literature demonstrating that endogenous electric fields of 30–200 mV/cm are present at wound sites and play physiological roles in guiding cell migration and neurite outgrowth during development and regeneration [15,16]. The electrodes were placed 2 cm apart, and the electric field uniformity was verified using a digital multimeter and COMSOL (Version 5.3) simulation. The culture medium pH and temperature were monitored during ES; no significant changes were observed (pH remained 7.35–7.45, temperature 37 ± 0.5 °C). Stimulation was administered once daily for 1 h, continuing consecutively over a 3-day duration. Control groups included: (1) GelMA/PCL (0% GO) with ES, (2) 1.0% GO without ES, and (3) 1.0% GO with ES.

### 2.10. Cell Viability, Attachment, and Morphology

After 3 days of culture, Cell viability and proliferation were quantitatively assessed using the CCK-8 assay (Cell Counting Kit-8, Beyotime, C0039, Shanghai, China). Briefly, 10 μL of the CCK-8 solution was added to each sample, and the absorbance at 450 nm was measured using a microplate reader (Bio-Rad Laboratories, iMark, China) after 2 h incubation, and viability (cytotoxicity) was calculated relative to the control group. Cells cultured on non-conductive GelMA/PCL nanofibrous scaffolds served as the control group. Viability is expressed as the mean ± standard deviation (SD) from three independent biological replicates (each with three technical replicates).

After 1 day of culture, PC12 morphology was investigated by SEM. Briefly, the samples were fixed with 2.5% (*v*/*v*) glutaraldehyde, dehydrated through a graded ethanol series (30%, 50%, 70%, 90%, 100%), completely dried in a critical point dryer (Leica, CPD 300, Wetzlar, Germany), and a layer of gold was sputtered onto the samples at a current of 40 mA for 60 s.

After 3 days of culture, PC12 cell morphology was examined using immunofluorescence staining. Briefly, cells were fixed with 4% paraformaldehyde for 30 min at room temperature, followed by permeabilization with 0.1% Triton X-100 for 10 min and blocking with 5% bovine serum albumin for 1 h. The samples were then incubated overnight with rabbit anti-βIII-βIII-tubulin antibody (1:1000, Abcam, Cambridge, UK) at 4 °C. After washing, the cells were incubated with rhodamine-conjugated goat anti-rabbit IgG (1:2000, Abcam) and DAPI (1:200, Life Technologies, Carlsbad, CA, USA) for 1 h and 5 min, respectively. The stained samples were examined using a confocal microscope (Leica TCS-SP8 STED 3X, Wetzlar, Germany) to visualize cell morphology and distribution. Due to dense neurite networks and scaffold autofluorescence, precise quantification of neurite length and angle was not performed; qualitative assessment is presented.

### 2.11. Statistical Analyses

All data are presented as the mean ± standard deviation (SD). Unpaired Student’s *t*-tests were used for two-group comparisons, and one-way or two-way ANOVA with Tukey’s post hoc test for multiple comparisons. *p* < 0.05 was considered significant. GraphPad Prism 9.0 was used.

## 3. Results

### 3.1. Synthesis and Characterization of Conductive GelMA/PCL/GO Nanofibrous Scaffolds

**Figure 1:**
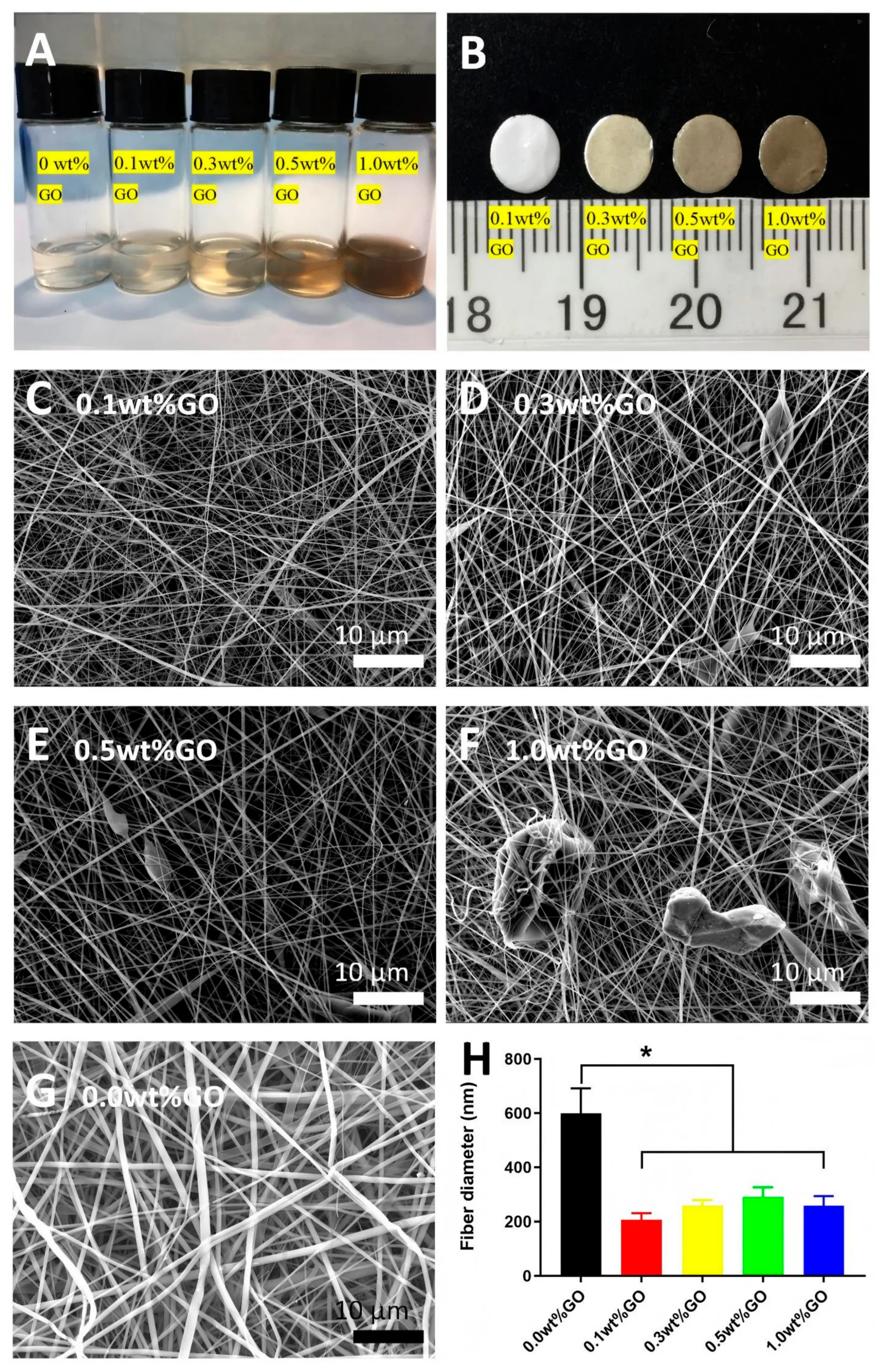
(**A**) The visual characteristics of the GelMA/PCL/GO hybrid exhibit a systematic evolution with increasing graphene oxide (GO) concentration, consistent with concomitant changes in light absorption behavior; all solutions remained macroscopically homogeneous, with no visible precipitation observed. (**B**) GelMA/PCL/GO nanofibrous scaffolds containing varying concentrations of graphene oxide (0.1, 0.3, 0.5, and 1.0 wt%). (**C**–**F**) Morphological characterization of GelMA/PCL/GO nanofibrous scaffolds with different GO concentrations: 0.1 wt% (**C**), 0.3 wt% (**D**), 0.5 wt% (**E**), and 1.0 wt% (**F**). Scale bar = 10 μm. (**G**) Morphology of GelMA/PCL nanofibrous scaffolds used as the control group. Scale bar = 10 μm. (**H**) Average fiber diameters of GelMA/PCL/GO nanofibrous scaffolds were quantified across varying graphene oxide (GO) concentrations—specifically 0% (Ctrl), 0.1% (**A**), 0.3% (**B**), 0.5% (**C**), and 1.0 wt% (**D**). * *p* < 0.05 compared to the control group. Error bars represent the standard deviation (SD).

As shown in Figure 1A, graphene oxide (GO) was dispersed in the GelMA/PCL spinning solution, demonstrating excellent colloidal stability. With increasing GO concentration (0.1–1.0 wt%), the solution gradually darkened—a trend consistent with the intrinsic optical absorption of GO. A corresponding increase in optical density was also observed in the resulting GelMA/PCL/GO nanofibrous scaffolds (Figure 1B). Fourier-transform infrared (FTIR) spectroscopy confirmed the successful incorporation of GO into the composite scaffolds. The characteristic absorption bands of PCL (1720 cm^−1^, C=O stretching; 1240 cm^−1^, C–O–C stretching) and GelMA (1640 cm^−1^, amide I; 1540 cm^−1^, amide II) were present in all scaffolds. The GO-containing scaffolds exhibited an additional broad band at 3200–3400 cm^−1^ (O–H stretching) and a distinct peak at 1620 cm^−1^ (aromatic C=C stretching), consistent with the oxygen-rich functional groups of GO (Supplementary Figure S1).

Raman spectroscopy provided unambiguous evidence for the presence of GO within the nanofibers. The characteristic D-band (~1350 cm^−1^) and G-band (~1590 cm^−1^) of graphitic carbon were clearly observed in GO-containing scaffolds (Supplementary Figure S2). The ID/IG ratio remained approximately 0.98, indicating that the electrospinning and UV crosslinking processes did not induce significant structural degradation of the GO nanosheets.

Scanning electron microscopy (SEM) analysis revealed that the composite nanofibers possessed smooth surfaces and formed an interconnected three-dimensional porous structure. SEM analysis of the scaffold surfaces revealed no macroscopic agglomeration; however, individual graphene oxide (GO) nanosheets could not be clearly distinguished due to insufficient electron contrast between GO and the polymer matrix (Figure 1C–H, Supplementary Figure S4). Notably, the diameters of the GelMA/PCL/GO nanofibrous scaffolds containing 0.1 wt%, 0.3 wt%, 0.5 wt%, and 1.0 wt% GO (200–300 nm) were significantly smaller than those of the GO-free GelMA/PCL nanofibers (~600 nm). However, further increasing the GO concentration did not yield a statistically significant change in fiber diameter (Figure 1C–H).

Thermogravimetric analysis (TGA) of the composite scaffolds showed a progressive increase in residual mass at 800 °C with increasing GO content, confirming successful GO incorporation at levels consistent with theoretical loading (Supplementary Figure S3).

### 3.2. Conductive Properties of GelMA/PCL/GO Nanofibrous Scaffolds

**Figure 2:**
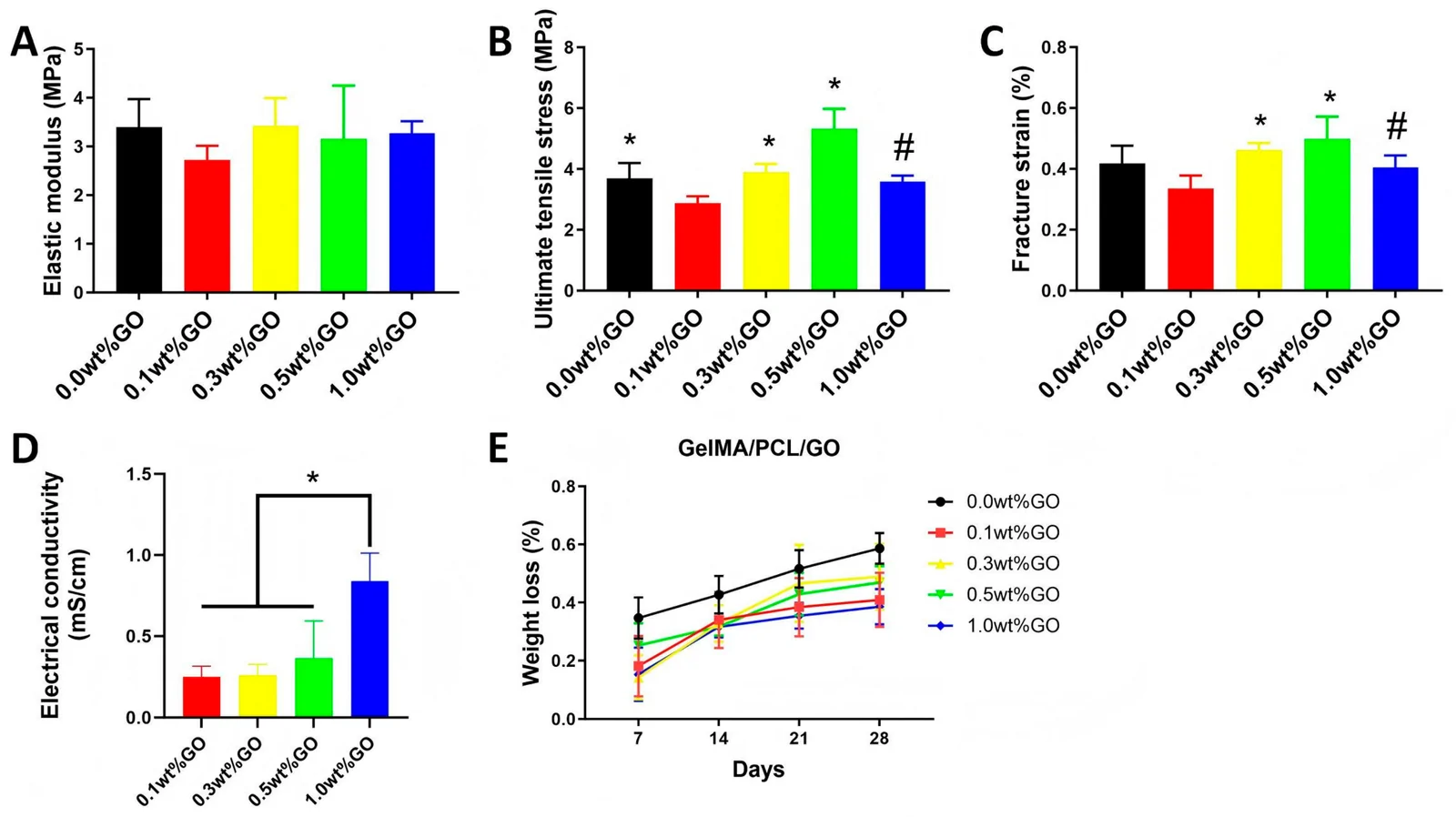
(**A**–**C**) Mechanical properties of GelMA/PCL/GO nanofibrous scaffolds with varying graphene oxide (GO) concentrations—0% (Ctrl), 0.1 wt% (**A**), 0.3 wt% (**B**), 0.5 wt% (**C**), and 1.0 wt% (**D**)—were assessed in terms of elastic modulus (**A**), fracture strain (**B**), and ultimate tensile stress (**C**). * *p* < 0.05 vs. the GelMA/PCL/0.1 wt% GO group; # *p* < 0.05 vs. the GelMA/PCL/0.5 wt% GO group. Error bars represent the SD. D) The electrical conductivity of GelMA/PCL/GO nanofibrous scaffolds containing graded concentrations of GO—0.1 wt% (A), 0.3 wt% (**B**), 0.5 wt% (**C**), and 1.0 wt% (**D**)—was measured to evaluate the effect of GO loading on conductive performance. (**E**) In vitro degradation profiles of GelMA/PCL/GO nanofibrous scaffolds with different GO contents—0% (Ctrl), 0.1 wt% (**A**), 0.3 wt% (**B**), 0.5 wt% (**C**), and 1.0 wt% (**D**)—were monitored over a 4-week immersion period in phosphate-buffered saline (PBS). * *p* < 0.05; Error bars represent the SD.

As shown in Figure 2D, incorporation of GO enhanced electrical conductivity. Conductivity values were 2.5 × 10^−4^ mS/cm (0.1 wt% GO), 2.6 × 10^−4^ mS/cm (0.3 wt% GO), 3.6 × 10^−4^ mS/cm (0.5 wt% GO), and 8.4 × 10^−4^ mS/cm (1.0 wt% GO). Statistical analysis revealed that only the 1.0 wt% GO group exhibited significantly higher conductivity than the 0.1 wt% GO group (*p* < 0.05), suggesting a percolation threshold effect rather than a linear or progressive increase. The maximum conductivity (8.4 × 10^−4^ mS/cm, equivalent to 8.4 × 10^−7^ S/cm) is modest but falls within the range reported for bioactive GO–polymer composites.

### 3.3. Hydrophilicity and Degradation Behavior of Conductive GelMA/PCL/GO Nanofibrous Scaffolds

Water contact angle measurements (Supplementary Video) revealed that all scaffolds—regardless of graphene oxide (GO) incorporation—exhibited superhydrophilic behavior, with contact angles approaching 0°. This wettability is primarily attributable to the intrinsic hydrophilicity of gelatin methacryloyl (GelMA) and the nanofibrous surface topography, rather than the presence of GO.

The in vitro degradation profiles exhibited significant variation among groups under both enzymatic and hydrolytic conditions (Figure 2E). Under enzymatic degradation with collagenase II, the GelMA/PCL group (Ctrl) showed moderate mass loss—approximately 59% by day 28—demonstrating that collagenase II effectively degrades the GelMA component, as expected from the literature. In contrast, all GO-containing groups exhibited significantly suppressed degradation, with the GelMA/PCL/1.0 wt% GO group displaying the lowest mass loss (approximately 38% by day 28). This suppression of degradation is likely attributable to the physical shielding of GelMA cleavage sites by GO nanosheets and to interfacial interactions that impede water penetration. Two-way repeated-measures ANOVA revealed a significant main effect of GO concentration (*p* < 0.01) and time (*p* < 0.0001). Post hoc analysis confirmed that the 1.0 wt% group had significantly lower weight loss than the control at day 28 (*p* < 0.05).

### 3.4. Mechanical Properties of Conductive GelMA/PCL/GO Nanofibrous Scaffolds

The elastic modulus remained unchanged upon GO incorporation (Figure 2A). Tensile strength and fracture strain increased with GO concentration up to 0.5 wt%, but decreased at 1.0 wt% GO to levels not significantly different from those of the control (0% GO) (Figure 2B,C). This indicates an optimal reinforcement effect at 0.5 wt% GO.

### 3.5. Cell Behavior on Conductive GelMA/PCL/GO Nanofibrous Scaffolds

**Figure 3:**
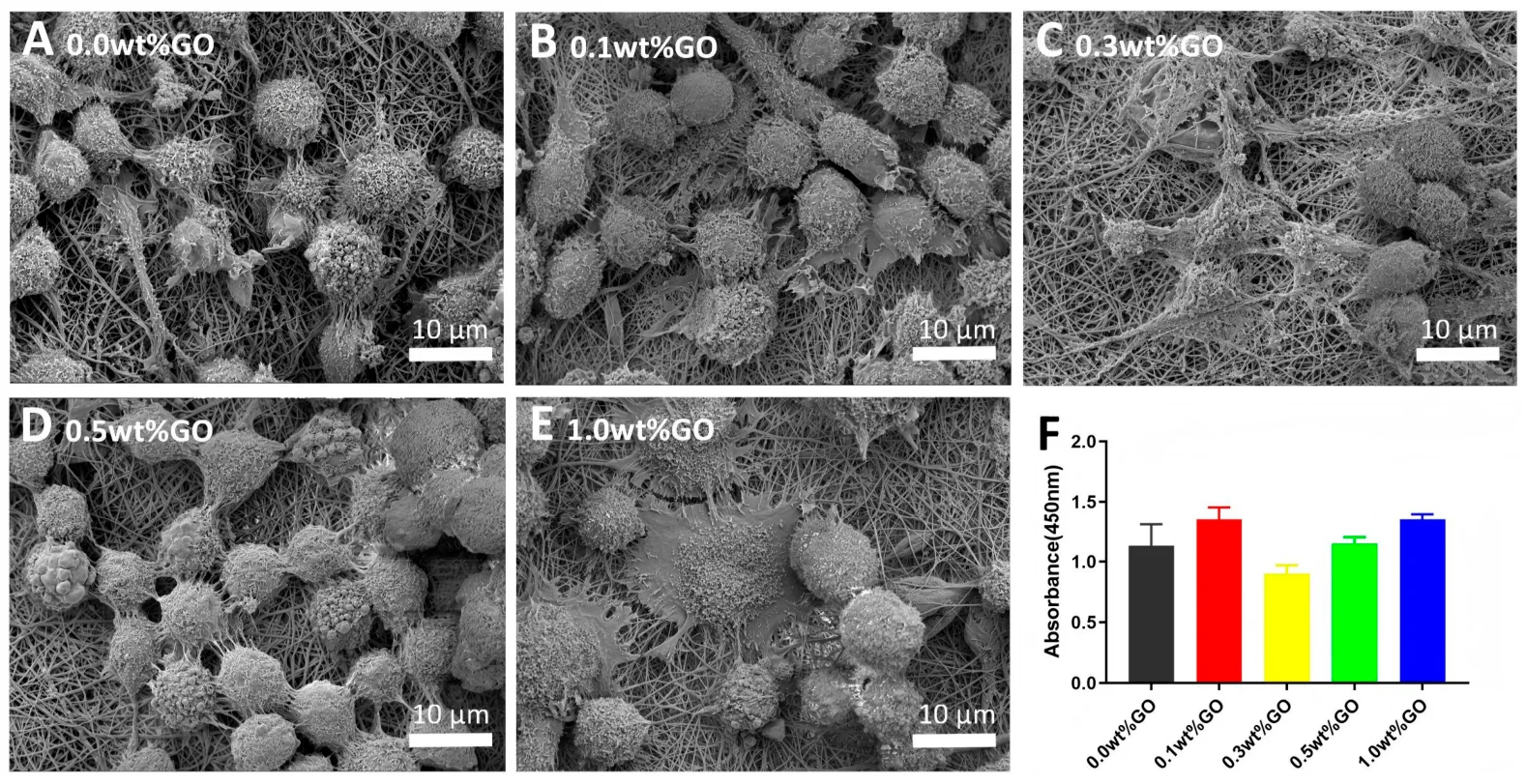
(**A**–**E**) SEM images of PC12 cells cultured on GelMA/PCL/GO nanofibrous scaffolds with varying GO concentrations: 0% (**A**), 0.1% (**B**), 0.3% (**C**), 0.5% (**D**), and 1.0 wt% (**E**); scale bar = 10 μm. (**F**) Quantitative analysis of cell proliferation after 72 h of culture. Error bars represent the SD, *n* = 3.

SEM images revealed firm cell adhesion and a well-spread morphology with filopodia (Figure 3A–E). The CCK-8 assay (Figure 3F) demonstrated that GO incorporation up to 1.0 wt% did not reduce cell viability.

**Figure 4:**
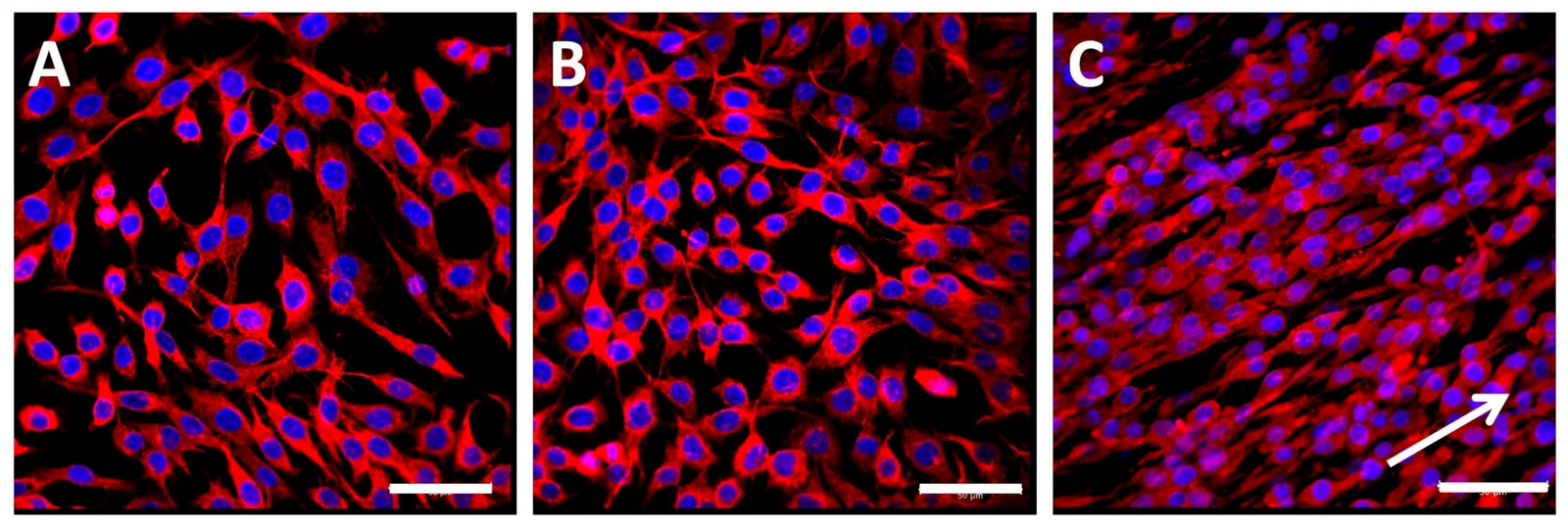
Enhanced cell spreading, axonal elongation, and directional alignment of PC12 cells were observed on GelMA/PCL nanofibrous scaffolds incorporating 1.0 wt% graphene oxide (GO) under electrical stimulation ((**C**), 1.0% GO with ES; the white arrow indicates the E-field direction), relative to both the non-stimulated control ((**B**), 1.0% GO without ES) and the GO-free GelMA/PCL scaffolds subjected to identical electrical stimulation ((**A**), GelMA/PCL (0% GO) with ES). βIII-tubulin (stained red) and nuclei (counterstained blue). Scale bar = 50 μm.

Immunofluorescence staining revealed qualitative differences. Under electrical stimulation (100 mV/cm), PC12 cells on the 1.0 wt% GO scaffold consistently exhibited a more elongated morphology and a visible tendency to align along the direction of the electric field (Figure 4C), whereas cells on the 0% GO scaffold with electrical stimulation or on the 1.0 wt% GO scaffold without electrical stimulation showed predominantly stellate or random orientations (Figure 4A,B). Precise quantification of neurite length and alignment angle was not feasible due to dense neurite networks and scaffold autofluorescence (see Discussion).

## 4. Discussion

The nervous system exhibits distinct electrophysiological features, making electrical signaling a critical factor in nerve injury repair [23,29]. Endogenous electrical signals play a pivotal role in tissue regeneration by guiding stem cell differentiation, modulating membrane enzyme activity, regulating ligand–receptor interactions, and inducing conformational changes in ion channels [30]. Hence, the optimization of electrical stimulation protocols for tissue engineering scaffolds remains an active area of research.

Graphene-based scaffolds—constructed from graphene, graphene oxide (GO), and reduced graphene oxide (rGO)—have shown significant promise in promoting peripheral nerve regeneration [7,8,9]. The choice between GO and rGO involves a trade-off between absolute conductivity and biological functionality. Our previous work used rGO [10]; here, we explore GO as an alternative. However, a direct experimental comparison of GO versus rGO with respect to protein adsorption or biochemical signaling was not performed in this study. This constitutes a limitation, and future work will address it.

In this study, we fabricated a series of electrospun conductive nanofibrous scaffolds incorporating systematically varied concentrations of graphene oxide (GO). Our results demonstrate that incorporation of 1.0 wt% GO significantly enhances electrical conductivity and promotes qualitative neurite elongation and alignment under applied direct-current electrical stimulation (100 mV/cm), whereas mechanical reinforcement—evidenced by tensile strength and elastic modulus—peaked at 0.5 wt% GO and declined at higher loading, likely due to incipient nanosheet agglomeration. Notably, scaffold hydrophilicity remained unchanged across all GO concentrations, indicating that the observed superhydrophilic behavior is attributable primarily to the intrinsic properties of GelMA and the nanofibrous topography, rather than to GO itself.

A critical consideration is the cytotoxicity profile. Dispersed GO nanoparticles can induce cytotoxicity [25,26], but when embedded in a polymer matrix, cellular exposure to GO is minimized. Our CCK-8 assay results indicate that scaffold-immobilized GO does not impair metabolic activity at concentrations up to 1.0 wt%, suggesting a favorable preliminary safety profile. However, a comprehensive assessment would require assays for reactive oxygen species (ROS), apoptosis, and inflammatory markers.

Mechanistic interpretation and hypothesis generation: Although this study demonstrates the physicochemical advantages and biocompatibility of GO-containing scaffolds—including enhanced electrical conductivity, improved PC12 cell viability, and ES-induced qualitative morphological changes—several mechanistic hypotheses remain to be experimentally validated within this specific system. These include (i) augmented protein adsorption mediated by GO’s oxygen-containing functional groups; (ii) modulation of cell–matrix interactions through GO-specific surface chemistry; and (iii) the precise contribution of scaffold conductivity to electrostimulation-triggered signal transduction pathways. All such proposals are grounded in established literature but have not been directly tested herein; they are therefore presented strictly as testable hypotheses to inform subsequent experimental work, not as empirically supported conclusions.

Fabrication yielded nanofibers with diameters of 200–300 nm—markedly smaller than those of pure GelMA/PCL scaffolds (~600 nm). The reduction in fiber diameter upon GO incorporation can be attributed to the increased electrical conductivity and charge density of the spinning solution, which enhance the stretching forces during electrospinning. The resulting nanoscale topography closely mimics the native extracellular matrix (ECM), providing an optimal microenvironment for cell adhesion, migration, and proliferation [17,18,31]. Raman mapping was attempted to visualize the spatial distribution of graphene oxide (GO); however, polymer autofluorescence severely compromised the signal-to-noise ratio, precluding reliable interpretation. SEM imaging was conducted exclusively on fiber surfaces—not on cross-sections—and confirmed the absence of large GO aggregates at the surface. AFM analysis was not viable due to topographical and mechanical constraints associated with the fibrous morphology. Consequently, while macroscopic surface agglomeration was effectively suppressed, the nanoscale dispersion of GO within the fiber bulk remains uncharacterized. Our inferences regarding GO’s influence on electrical conductivity and cellular responses are therefore grounded in bulk-level observations—specifically, a percolation-like enhancement in conductivity and the lack of surface-scale aggregates—rather than direct experimental evidence of nanoscale homogeneity. Complementary techniques such as cryo-TEM of ultramicrotomed cross-sections or HAADF-STEM with heavy-metal staining represent promising avenues for future work to resolve GO localization and distribution at the nanoscale.

Electrical conductivity increased significantly only at 1.0 wt% GO, reaching 8.4 × 10^−4^ mS/cm (8.4 × 10^−7^ S/cm). This value is modest; we do not claim that it matches native nerve conductivity. Instead, we propose that the scaffold acts as a substrate to locally transmit the externally applied electric field (100 mV/cm), which serves as the primary driver of cellular response. This interpretation aligns with several recent studies on low-conductivity polymer scaffolds for neural stimulation [32].

Notably, GO is uniformly dispersed and fully encapsulated within the insulating polymer matrix, thereby effectively suppressing uncontrolled current leakage and mitigating potential electrochemical side reactions. Furthermore, the applied field strength (100 mV/cm) falls within the physiological range observed in endogenous wound electric fields, and no measurable deviations in local pH or temperature were detected during stimulation. Collectively, these findings indicate that the electroactive scaffold presents minimal risk of exacerbating pathological electrical discharge; however, rigorous in vivo evaluation—including longitudinal monitoring of local tissue impedance and electrochemical biocompatibility—is warranted in subsequent studies.

Biological assessments showed that PC12 cells attached and proliferated well on all scaffolds. Under electrical stimulation, the 1.0 wt% GO scaffold promoted qualitative neurite elongation and alignment. This effect was specific to the combination of GO and ES, as neither 0% GO with ES nor 1.0% GO without ES produced comparable morphological changes. However, the use of PC12 cells (a cell line) does not fully recapitulate primary neurons or the in vivo environment, which includes Schwann cells, macrophages, and other support cells.

A technical limitation of this study is the inability to perform precise morphometric quantification of neurite length or alignment angle. PC12 cells on electrospun scaffolds formed dense, overlapping neurite networks that prevented unambiguous tracing of individual neurites. Additionally, the electrospun GelMA/PCL/GO scaffold exhibited mild autofluorescence in the same spectral range as the βIII-tubulin antibody, thereby reducing the signal-to-noise ratio for fine distal neurites in conventional epifluorescence images. Future studies employing confocal z-stack acquisition, deconvolution, and semi-automated tracing algorithms (e.g., NeuronJ or Neurolucida) using primary neurons cultured on flat conductive films or on scaffolds with reduced autofluorescence will be necessary to rigorously quantify neurite elongation and alignment. Therefore, the morphological observations presented here should be considered qualitative and hypothesis-generating.

Several other limitations should be acknowledged: (1) PC12 cells are a model cell line; future studies should incorporate primary dorsal root ganglion neurons and Schwann cells. (2) The electrical stimulation protocol (100 mV/cm, direct current) is simplified; alternating current or biphasic stimulation may better mimic physiological conditions. (3) Transmission electron microscopy (TEM) or higher-resolution imaging would be required to definitively confirm GO dispersion. (4) No in vivo evaluation was performed; this is a preliminary in vitro study. (5) A comprehensive cytotoxicity assessment—including reactive oxygen species (ROS) generation, apoptosis, and inflammatory marker expression—is required to establish safety.

## 5. Conclusions

In this preliminary in vitro study, we successfully fabricated a conductive GelMA/PCL/graphene oxide (GO) nanofibrous scaffold that combines a biomimetic architecture with enhanced electroactivity and excellent biocompatibility. The 1.0 wt% GO composite was identified as the optimal formulation for electrical conductivity and for qualitatively promoting neurite elongation and alignment under applied direct-current electrical stimulation (100 mV/cm), although mechanical reinforcement peaked at 0.5 wt% GO. These results indicate that the scaffold holds promise as a nerve guidance conduit (NGC), pending further validation using primary neurons, a comprehensive safety assessment, and in vivo studies in a rodent sciatic nerve defect model.

## Data Availability

All original experimental data supporting the findings of this study are available from the corresponding author upon reasonable request. The data will be deposited in a public open-access repository after the manuscript is formally published.

## Supplementary Materials

The following supporting information can be downloaded at https://www.mdpi.com/article/10.3390/jfb17080376/s1, Supplementary Figure S1: FTIR spectra of GO and GelMA/PCL/GO scaffolds; Supplementary Figure S2: Raman spectra of graphene oxide (GO) and GO incorporated into GelMA/PCL scaffolds containing 1.0 wt% GO; Supplementary Figure S3: TGA curves of scaffolds with varying GO content; Supplementary Figure S4: Morphological characterization of GelMA/PCL/GO nanofibrous scaffolds as a function of graphene oxide concentration (scanning electron microscopy images acquired at lower magnification); Supplementary Video: Water contact angle measurements.
