# Supplementary material for: In Vitro Evaluation of Electroactive GelMA/PCL/Graphene Oxide Scaffolds for Peripheral Nerve Repair
Source: J Funct Biomater. 2026 Aug 3;17(8):376. doi: 10.3390/jfb17080376 (PMC13514470; doi:10.3390/jfb17080376)
Supplement: Supplementary file 1 [file jfb-17-00376-s001.zip › jfb-4313004-supplementary.pdf]

Supplementary Information

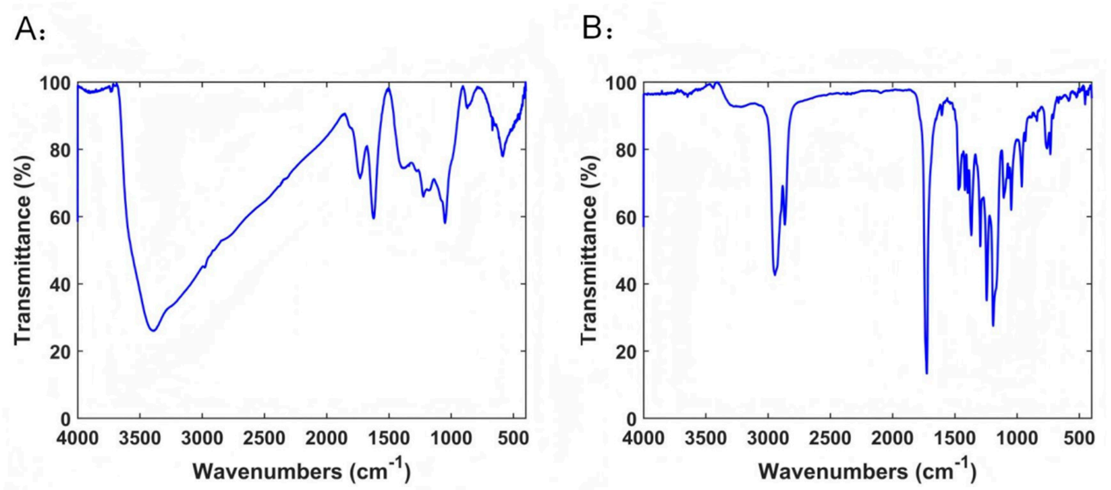

Figure S1: FTIR spectra of GO (A) and GelMA/PCL/GO (B) scaffolds.

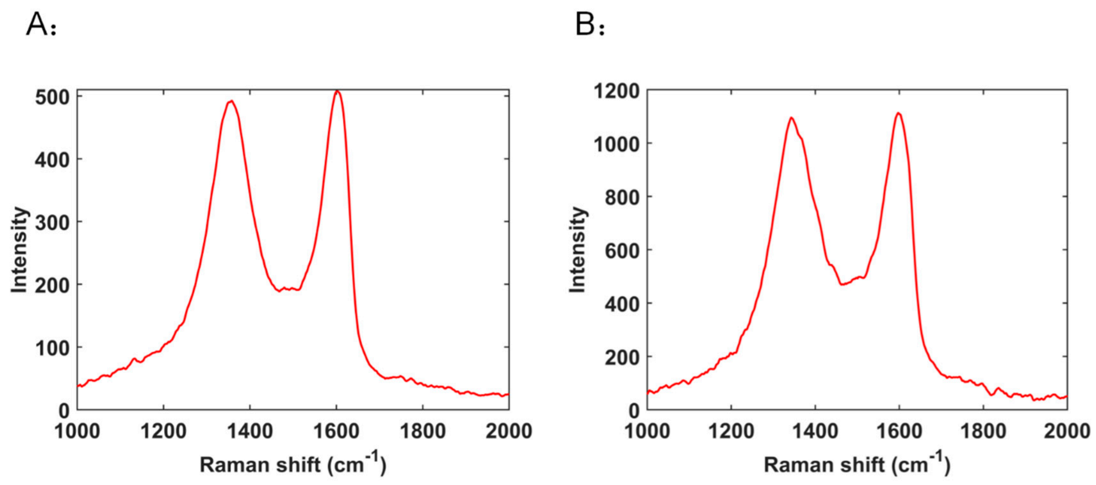

Figure S2: Raman spectra of graphene oxide (GO) (A) and GO incorporated into GelMA/PCL scaffolds containing 1.0 wt% GO (B).

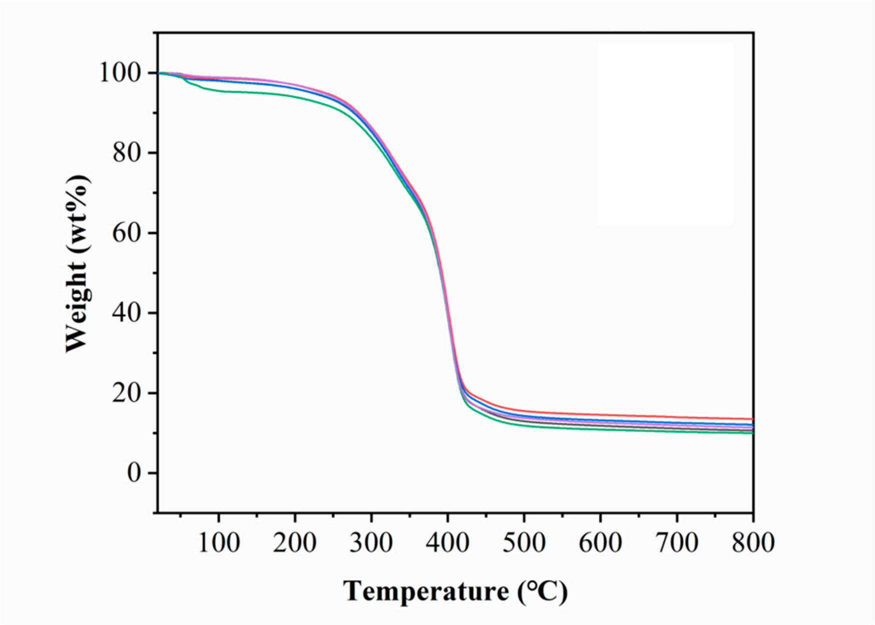

Figure S3: TGA curves of scaffolds with varying GO content. GelMA/PCL/GO nanofibrous scaffolds with different GO contents—0% (green), 0.1 wt% (black), 0.3 wt% (purple), 0.5 wt% (blue), and 1.0 wt% (red).

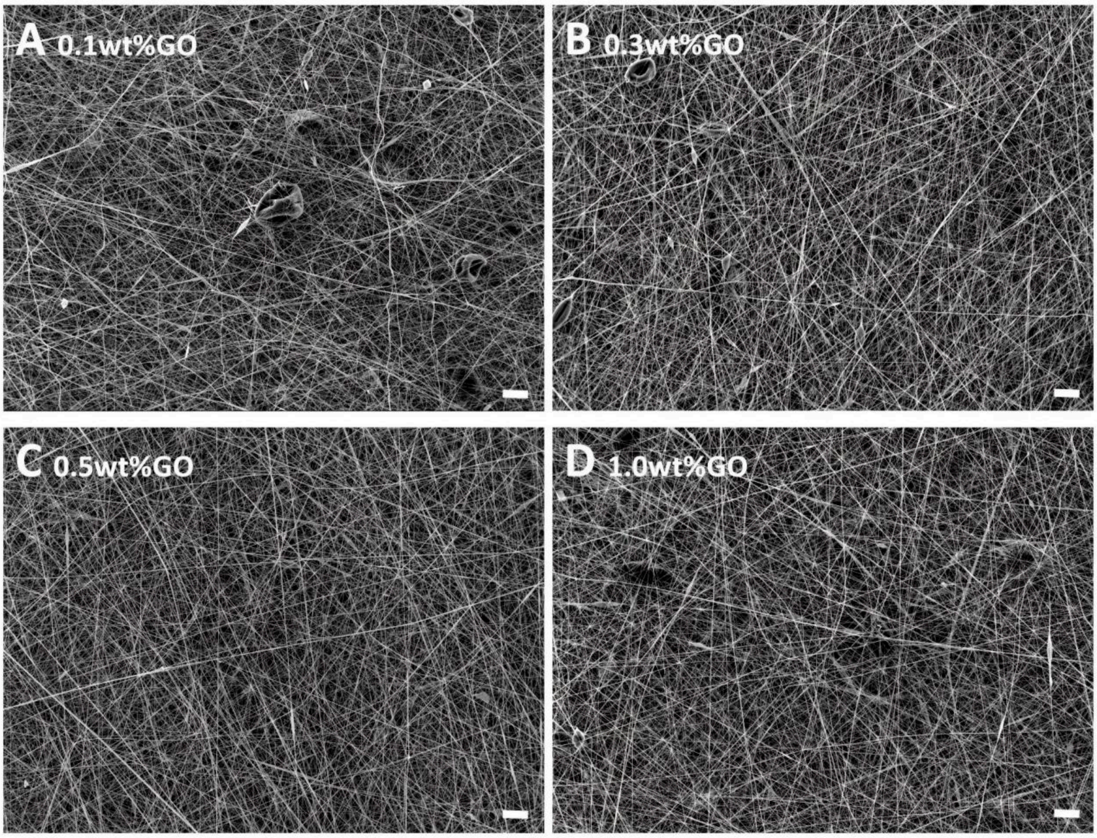

Figure S4: Low-magnification scanning electron microscopy (SEM) images of GelMA/PCL/GO nanofibrous scaffolds prepared with systematically varied graphene oxide (GO) concentrations: 0.1

wt% (A), 0.3 wt% (B), 0.5 wt% (C), and 1.0 wt% (D). These images were acquired from the same scaffold batches as those shown in Figure 1(C–F), using reduced magnification to support rigorous, quantitative evaluation of surface homogeneity across extended fields of view. Critically, Figure 1(D) and Supplementary Figure S4(B) were captured from spatially non-overlapping imaging fields within the same 0.3 wt% GO scaffold sample; they represent independent acquisitions and are neither cropped nor digitally magnified derivatives of a common micrograph. Scale bars = 10 μm.
